# Antifungal effects and biocontrol potential of lipopeptide-producing *Streptomyces* against banana Fusarium wilt fungus *Fusarium oxysporum* f. sp. *cubense*

**DOI:** 10.3389/fmicb.2023.1177393

**Published:** 2023-04-27

**Authors:** Xiaxia Wang, Zhenghua Du, Chanxin Chen, Shuang Guo, Qianzhuo Mao, Wei Wu, Ruimei Wu, Wenbo Han, Peifeng Xie, Yiping Zeng, Wenna Shan, Zonghua Wang, Xiaomin Yu

**Affiliations:** ^1^State Key Laboratory of Ecological Pest Control for Fujian and Taiwan Crops, College of Plant Protection, Fujian Agriculture and Forestry University, Fuzhou, China; ^2^FAFU-UCR Joint Center for Horticultural Biology and Metabolomics, Haixia Institute of Science and Technology, Fujian Agriculture and Forestry University, Fuzhou, China; ^3^School of Life Sciences, Fujian Agriculture and Forestry University, Fuzhou, China; ^4^College of Horticulture, Fujian Agriculture and Forestry University, Fuzhou, China; ^5^State Key Laboratory for Managing Biotic and Chemical Threats to the Quality and Safety of Agro-Products, Institute of Plant Virology, Ningbo University, Ningbo, China; ^6^Fujian Universities Engineering Research Center of Marine Biology and Drugs, Fuzhou Institute of Oceanography, Minjiang University, Fuzhou, China

**Keywords:** antifungal effect, *Streptomyces*, banana Fusarium wilt, lipopeptide, biocontrol

## Abstract

Fusarium wilt of banana (FWB), caused by *Fusarium oxysporum* f. sp. *cubense* (Foc), especially tropical race 4 (TR4), presents the foremost menace to the global banana production. Extensive efforts have been made to search for efficient biological control agents for disease management. Our previous study showed that *Streptomyces* sp. XY006 exhibited a strong inhibitory activity against several phytopathogenic fungi, including *F. oxysporum*. Here, the corresponding antifungal metabolites were purified and determined to be two cyclic lipopeptide homologs, lipopeptin A and lipopeptin B. Combined treatment with lipopeptin complex antagonized Foc TR4 by inhibiting mycelial growth and conidial sporulation, suppressing the synthesis of ergosterol and fatty acids and lowering the production of fusaric acid. Electron microscopy observation showed that lipopeptide treatment induced a severe disruption of the plasma membrane, leading to cell leakage. Lipopeptin A displayed a more pronounced antifungal activity against Foc TR4 than lipopeptin B. In pot experiments, strain XY006 successfully colonized banana plantlets and suppressed the incidence of FWB, with a biocontrol efficacy of up to 87.7%. Additionally, XY006 fermentation culture application improved plant growth parameters and induced peroxidase activity in treated plantlets, suggesting a possible role in induced resistance. Our findings highlight the potential of strain XY006 as a biological agent for FWB, and further research is needed to enhance its efficacy and mode of action *in planta*.

## Introduction

Banana (*Musa* spp.) is the most traded fruit in the world with ~140 million metric tons produced in 2021 (FAOSTAT, 2021), providing food security and income to over 400 million people globally. More than 40% of world banana production is based on the cultivars of the Cavendish sub-group (AAA), which make up almost all the export trade (Dale et al., 2017). The monoculture practice based on a single variety has made banana cultivation very vulnerable to pests and diseases (Ghag et al., 2015). Among these diseases, Fusarium wilt of banana (FWB), a widespread devastating soil-borne disease caused by *Fusarium oxysporum* f. sp. *cubense* (Foc), poses a major constraint on banana production worldwide (Dale et al., 2017).

The known Foc strains were classified into four races according to host specificity, with race 4 later being subdivided into subtropical race 4 (STR4) and tropical race 4 (TR4; Nguyen et al., 2019). Foc TR4, also known as *F. odoratissimum*, is the most destructive haplotype that causes FWB in Cavendish cultivars as well as other important genotypes in tropical and subtropical regions (Butler, 2013; Ploetz, 2015; Maryani et al., 2019). First identified in Taiwan in 1967, Foc TR4 has spread at an alarming rate to more than 20 banana-growing countries in Asia, Africa and more recently in Latin America, wreaking havoc to the global banana industry (van Westerhoven et al., 2022). FWB is exceedingly difficult to manage as Foc chlamydospores could persist in soil for several decades; moreover, non-host plant species (e.g., weeds) that are colonized endophytically by Foc strains may act as reservoirs for pathogen dissemination (Pegg et al., 2019). The use of synthetic chemicals is a common practice in the field to manage Foc TR4, but only shows limited efficacy (Getha and Vikineswary, 2002). Besides, extensive use of chemical fungicides may pose risks to environment and human health. Other management practices that have been implemented include the development of resistant varieties, field sanitation, crop rotation, application of biological agents and organic amendments, and the use of cover crops (Dita et al., 2018). Among all the methods, biological control has received the widest attention as an effective and sustainable strategy to suppress plant disease and improve crop productivity (Bubici et al., 2019).

Studies have shown that certain groups of microorganisms, such as *Trichoderma* (Tao et al., 2023), *Pseudomonas* (Lv et al., 2023), *Bacillus* (Xiang et al., 2023), and *Streptomyces* (Zhu et al., 2023), are effective against Foc. In fact, these microorganisms have been recorded to significantly reduce disease incidence and severity caused by Foc TR4 in greenhouse and field trials (Bubici et al., 2019), suggesting that they have a great potential to be developed as promising biocontrol agents (BCAs). The genus *Streptomyces* is renowned for producing an arsenal of specialized metabolites with diverse bioactivities (Barka et al., 2015). One of the key features contributing to its success as effective BCAs is the production of an impressive array of metabolites and enzymes with antimicrobial activities towards various plant phytopathogens and as such this genus has been widely used in crop protection (Doumbou et al., 2001). In addition to acting as BCAs, *Streptomyces* species, especially those isolated from plants (rhizosphere and endosphere), possess a widespread ability to promote plant growth and contribute to plant stress alleviation through multifactorial mechanisms (Pang et al., 2022). Despite the good application prospect, streptomycetes in general have been less evaluated compared to other microbial taxa against Fusarium diseases and few products based on *Streptomyces* and their bioactive molecules are available in the market (Bubici, 2018). Therefore, more efforts are required to explore the potential of this prominent group of antibiotic producers for FWB antagonism.

In our previous study, we demonstrated that endophytic *Streptomyces* sp. XY006 isolated from the tea plant exhibited a good inhibitory effect against multiple phytopathogenic fungi *in vitro*, such as *F. oxysporum*, *Magnaporthe grisea*, and *Colletotrichum* sp., among which *F. oxysporum* was most obviously inhibited (Shan et al., 2018). However, compounds accountable for the antifungal activity and their potential modes of action are not clear. Therefore, in this study, we reported the isolation and purification of the antifungal metabolites of strain XY006 and evaluated their antifungal effects against Foc TR4 *in vitro*. In parallel, we also investigated the colonization dynamics and the biocontrol efficiency of strain XY006 against FWB by pot experiments. Taken together, our results demonstrate that strain XY006 has a significant potential in the control of FWB.

## Materials and methods

### Strains and culture conditions

Endophyte *Streptomyces* sp. XY006, isolated previously from the leaves of a tea plant (Shan et al., 2017), was cultured on ISP2 agar (Difco; 1% malt extract, 0.4% yeast extract, 1% glucose, and 2% agar) at 30°C. Foc TR4 was maintained on complete medium (Correll, 1987) or potato dextrose broth (PDB, Merck; Yang et al., 2021) at 28°C.

### Isolation and purification of antifungal compounds from *Streptomyces*

To obtain antifungal compounds, 100 mL MYG (1% malt extract, 0.4% yeast extract, and 1% glucose) broth was inoculated with 1 mL starter culture of strain XY006 and agitated on a rotary shaker at 200 rpm for 5 days at 30°C. The culture was then plated onto 14 L mannitol soya flour (MS) agar plates (2% mannitol, 2% soya flour, and 2% agar) and incubated at 30°C for additional 7 days. Extract was harvested by freezing the plates at − 80°C, followed by thawing and squeezing to liberate the liquid. The obtained water-soluble fraction was then concentrated by rotary evaporation and extracted with 70% (*v*/*v*) methanol. After centrifugation, the supernatant was again evaporated *in vacuo*, resuspended in water and extracted twice with an equal volume of *n*-butanol. The butanol layer was evaporated to dryness to give 10.7 g of crude extract. This crude extract, after dissolved in water, was loaded onto one OASIS HLB cartridge (Waters, 6 cc, 200 mg) preconditioned with 2 mL methanol and 2 mL water. Once the sample passed through the cartridge, stepwise gradients of methanol and water (0:100, 20:80, 40:60, 60:40, 80:20, and 100:0) were applied for elution, yielding six fractions. After concentration *in vacuo*, the antifungal activity of each fraction was measured against Foc TR4 by a paper disc diffusion assay (Mearns-Spragg et al., 1998), with a few modifications. Briefly, a small square (2 mm × 2 mm) was cut from the mycelial edges of Foc TR4 and inoculated into the center of complete medium plates. Sterile filter paper discs were placed 2 cm away from the Foc TR4 cuts and 2 mg of bioactive compounds were added to each disc. The plates were then incubated at 30°C for 3–5 days to observe the antifungal activities. The 60% methanol fraction (290 mg), which showed a high antifungal activity, was further fractionated by preparative high-performance liquid chromatography (HPLC; Waters Prep 150 LC System, United States) with an XBridge Prep OBD C_18_ column (5 μm, 19 mm × 150 mm). Water and methanol were used as the mobile phases A and B, respectively, at a flow rate of 5 mL/min. The gradient used was as follows: 0–0.1 min (50% B), 0.1–15 min (50–100% B) and 15–25 min (held at 100% B). The photo diode array detector was set to the wavelength range of 200–300 nm. Each fraction (5 mL) was collected using a fraction collector. Their antifungal activities against Foc TR4 were assessed using the aforementioned paper disc diffusion assay. Fractions 18–21 (126 mg in total) showing the antifungal activity were pooled and further purified on the same column using a linear gradient from 50% to 100% acetonitrile over 15 min at a flow rate of 5 mL/min. Finally, antifungal compounds SL-1 (8.2 mg) and SL-2 (13.1 mg) were obtained from fractions 13 and 9, respectively. The remaining fractions, which contained co-purified SL-1 and SL-2 (89.2 mg and 90% combined purity), were pooled and lyophilized.

### Mass spectrometry analysis of the purified antifungal compounds

The purified antifungal metabolites (SL-1 and SL-2) were analyzed on an ultra-performance liquid chromatography (UPLC) hyphenated to quadrupole time-of-flight mass spectrometer (Synapt G2-Si HDMS, Waters, Manchester, United Kingdom; UPLC-QTOFMS) equipped for electrospray ionization. The chromatography was performed at a flow rate of 0.3 mL/min on a Waters Acquity UPLC HSS T3 column (100 mm × 2.1 mm, 1.8 μm) using 0.1% formic acid (phase A) and acetonitrile containing 0.1% formic acid (phase B) as the mobile phase. The gradient used was as follows: 0–5 min (50%–100% B), 5–8 min (held at 100% B), 8–8.1 min (100–50% B) and allowed to equilibrate for a further 2 min prior to the next injection. The injection volume was 1 μL. A full scan with positive ionization mode was conducted from *m*/*z* 50 to 1,500 with a cone voltage of 40 V, capillary voltage of 1.28 kV and desolvation temperature of 350°C. The MS data were acquired in the continuum mode using ramp collision energy from 10 to 50 eV. MS/MS fragmentation was applied to the selected precursor ions in positive ionization mode with a cone voltage of 80 V, capillary voltage of 2 kV and desolvation temperature of 350°C.

### Inhibition of Foc TR4 mycelial growth by purified antifungal compounds

Given the limited amount of pure SL-1 and SL-2 obtained, a combined treatment with co-purified SL-1 and SL-2 complex (referred hitherto as “SL”) was applied unless otherwise stated. SL, SL-1, and SL-2 were dissolved in sterile pure water. To examine the inhibitory effect on Foc TR4 mycelial radial growth, SL was amended to the autoclaved complete medium for a final concentration of 50 or 500 μg/mL. Blank medium extract (50 μg/mL), which was obtained from MS agar plates subjected to the same purification procedures as SL crude extracts, was used as a negative control. A small square (2 mm × 2 mm) of Foc TR4 grown on complete medium was cut from the margin of the colony and placed in the center of a fresh complete medium plate. The diameter of the colony was measured 3 days after incubation at 28°C. Growth inhibition was calculated using the following formula: Inhibition rate (%) = [(*C*−*T*)/*C*] × 100, where *C* and *T* are the diameters of the colony on the negative control and the SL-amended medium, respectively. In the meantime, the mycelial dry weight was determined. The experiments were performed in triplicate with three plates per replicate.

### Inhibition of Foc TR4 sporulation by purified antifungal compounds

Foc TR4 spore suspensions (10^6^ conidia/mL) derived from complete medium plates were used to inoculate (1% v/v) potato dextrose broth (PDB) supplemented with 50 or 500 μg/mL SL or 50 μg/mL blank medium extract (negative control). To compare the effects of lipopeptin A and lipopeptin B on spore inhibition, these two compounds were also individually supplemented to PDB at final concentrations of 5, 10, 25, 50, 100, 200, and 500 μg/mL, respectively. After cultivation at 28°C for 3 days, conidia were collected and filtered through four layers of lens cleaning paper to remove mycelial debris. The number of conidia was counted using a hemocytometer under a light microscope. All experiments were performed in triplicate. The half-maximal inhibitory concentration (EC50) values of SL-1 and SL-2 were calculated in GraphPad Prism 8.

#### Changes in the fatty acid content of Foc TR4 cell membrane induced by purified antifungal compounds

SL at a final concentration of 500 μg/mL was added to the complete medium of Foc TR4 precultivated for 14 h. Culture treated with only blank medium extract was set as the control group. Following cultivation for another 24 h, cells were pelleted down (8,000*g*, 10 min), washed three times with sterile water and air-dried. Approximately 100 mg of fungal cells was added to a round bottom flask prefilled with 100 mL methanol. The reaction mixture was refluxed for 2 h and then cooled down to room temperature. The reflux was concentrated to 2 mL *in vacuo* and hydrolyzed with 11 g/L sodium hydroxide at 90°C for 10 min. Total fatty acids were derivatized with 2 mL methanolic sulfuric acid (10% *v*/*v*), 75 μg of margaric acid (internal standard), 25 μL 0.2% butylated hydroxytoluene in methanol (*w*/*v*) and 300 μL toluene at 90°C for 20 min. After cooling down to room temperature, the fatty acid solution was extracted with 1.5 mL 0.9% sodium chloride and 2 mL hexane. The hexane layer was dried over anhydrous sodium sulfate, concentrated *in vacuo* and transferred to a 2 mL GC vial. Fatty acid analysis was performed with GCMS-QP2010 Ultra Gas Chromatograph Mass Spectrometer (Shimadzu, Kyoto, Japan) equipped with a Restek RT 2560 capillary column and a frame ionization detector as previously described (Guo et al., 2020). All samples were analyzed in triplicate. The relative abundance of individual fatty acid was expressed as the proportion of the sum of all fatty acids.

#### Changes in Foc TR4 ergosterol content induced by purified antifungal compounds

To 100 mg of Foc TR4 cells in a sealed vial, 5 mL potassium hydroxide solution (5 g potassium hydroxide, 5 mL sterile distilled water, and 95 mL methanol) and 10 μg of 5*α*-cholestane (internal standard) were added. The mixture was reacted at 70°C for 1 h, cooled down to room temperature and extracted twice with an equal volume of hexane. The hexane layer was dried over anhydrous sodium sulfate and transferred to a 2 mL GC vial, followed by derivatization with *N*,*O*-bis(trimethylsilyl)trifluoroacetamide +1% trimethylchlorosilane (BSTFA + 1% TMCS) at 80°C for 45 min. The quantitative analysis of ergosterol was accomplished on an Agilent 7890B Gas Chromatograph, which was equipped with a Restek Rxi®-5Sil MS capillary column (30 m × 0.25 mm × 0.25 μm) and coupled to Pegasus HT time-of-flight mass spectrometer (LECO, St. Joseph, MI, United States; GCTOF-MS). Helium was used as the carrier gas with a flow rate of 1.5 mL/min. One μL sample was injected in the splitless mode at an inlet temperature of 280°C. The MS transfer line temperature was maintained at 275°C. Oven temperature started at 170°C for 1.5 min, ramped to 280°C at 37°C/min, and increased to 300°C at 1.5°C/min, and retained for 5 min. Ergosterol was quantified by peak area relative to an external calibration curve by the method as described earlier (Zhou et al., 2019) and expressed as milligrams per gram of mycelium (fresh weight). All samples were analyzed in triplicate.

#### Changes in Foc TR4 mycelial morphology induced by purified antifungal compounds

Foc TR4 was cultured on complete medium plates amended with SL or blank medium extract (negative control) for 3 days according to the aforementioned method. A small square (2 mm × 2 mm) cut from the mycelial edges was prefixed with 4% glutaraldehyde containing 1% Triton X-100 for 3 days, rinsed with 0.1 M sodium phosphate buffer (pH 7.0) three times (every time for 15 min) and fixed in 1% osmic acid for 2 h. Following three successive washes by 0.1 M sodium phosphate buffer, samples were dehydrated in alcohol (50, 70, 80, 90, 95, and 100%, for 15 min each), dried using a Leica CPD300 critical point dryer (Germany) and sputter-coated with gold. Afterward, specimens were observed under a Hitachi SU3500 Scanning Electron Microscope (SEM; Tokyo, Japan).

#### Changes in Foc TR4 mycelial ultrastructure induced by purified antifungal compounds

Methods for sample treatment, fixation and dehydration were the same as described above. Samples were embedded in Spurr resin overnight and re-embedded for aggregation at 70°C for 24 h. The embedded blocks were sectioned with an ultramicrotome (Leica EM UC7, Germany), stained in uranyl acetate for 15 min and lead citrate solution for 10 min. Finally, samples were observed under a Hitachi HT7800 Transmission Electron Miscroscope (TEM; Tokyo, Japan).

#### Changes in Foc TR4 fusaric acid (FA) production induced by purified antifungal compounds

The aforementioned Foc TR4 spore suspensions were used to inoculate (1% v/v) 5 mL Czapek medium supplemented with 500 μg/mL SL or 50 μg/mL blank medium extract (negative control). After culturing for 15 days at 28°C, the culture medium was acidified to pH 3.0 with hydrochloric acid and extracted with 5 mL ethyl acetate. The organic phase thus obtained was evaporated to dryness and derivatized with 100 μL BSTFA +1% TMCS at 70°C for 30 min. The quantitation of FA was performed through Agilent 7890B Gas Chromatograph and LECO HT time-of-flight mass spectrometer equipped with a Restek Rxi®-5Sil MS capillary column (30 m × 0.25 mm × 0.25 μm) as described above. Helium was used as the carrier gas with a flow rate of 1.5 mL/min. Oven temperature started at 70°C for 2 min, increased to 280°C at 10°C/min, and retained for 2 min. FA in fungal samples was quantified by peak area relative to an external calibration curve constructed using a commercial FA standard and expressed as milligrams per gram of mycelium (dry weight). All samples were analyzed in triplicate.

### Colonization dynamics of banana seedlings by strain XY006

To explore the colonization potential of strain XY006 on banana seedlings, we tagged this strain with the enhanced GFP (eGFP) marker. Briefly, the gene encoding eGFP was cloned into the pIB139 vector (Wilkinson et al., 2002), which carries the ermE* promoter, an apramycin resistance marker and a lambda phage chromosomal integration sequence. Conjugal transfer of plasmid pIB139-*eGFP* from *Escherichia coli* donor strain ET12567/pUZ8002 to strain XY006 was established following the standard protocol (Kieser et al., 2000). Positive ex-conjugants were selected on the basis of apramycin resistance and the presence of *eGFP* gene was confirmed by PCR. The expression of *eGFP* was examined using an inverted microscope (Nikon ECLIPSE TI-E, Kobe, Japan). The transformed XY006 strain (t-XY006) was grown on MS agar plate at 30°C for 10 days to prepare spore stocks for plant inoculation.

The tissue cultured seedlings of Cavendish banana (AAA cultivar) were carefully removed from seedling bags. Roots were thoroughly rinsed with sterile water and soaked in t-XY006 spore suspensions (10^6^ CFU/mL) with gentle shaking for 20 h. Seedlings were dried under the laminar flow hood and transplanted into plastic pots (6.5 cm × 6.5 cm) filled with a mixture of autoclaved peat soil and vermiculite in 1:1 ratio, and maintained in a growth chamber (27°C, 78% relative humidity and 12 h photoperiod).

To understand the colonization dynamics, the t-XY006 strain was re-isolated 4, 7, 11, 14, 21, and 28 days post inoculation (dpi) from root, corm and leaf tissues of five banana seedlings according to the following procedures. The entire seedling was carefully detached from the growth substrate and the bulk soil was removed by gentle shaking. The cleaned roots, corms and leaves were cut from the seedlings. They were surface-sterilized sequentially using: 70% (*v*/*v*) ethanol for 1 min, sterile water five times with 1 min each, sodium hypochlorite for 30 s and sterile water five times with 1 min each. The water from the final rinse was plated onto LB plates to verify the absence of contaminants. Subsequently, these surface-sterilized tissues were finely homogenized with sterile water using a sterile mortar and the suspensions were plated in serial dilutions on MS agar plates supplemented with 50 μg/mL apramycin. Plates were incubated at 30°C and streptomycete colonies were streaked for purity. They were subjected to 16S rRNA sequencing to confirm the identity. The t-XY006 concentration was expressed as CFU/g of fresh weight in respective tissues.

### Assessment of the biocontrol efficacy of strain XY006 against FWB under growth chamber conditions

Biological activity of XY006 against Foc TR4 was investigated in a growth chamber (28°C, 78% relative humidity and 12 h photoperiod) using plastic pots filled with the growth substrate as mentioned above. Banana plantlets with five to six leaves were selected to conduct the pot experiments. The pots inoculated with only Foc TR4 were used as the inoculated control. The pots inoculated with neither Foc TR4 nor XY006 were used as the non-inoculated control. XY006 was cultured in MYG broth and incubated at 30°C with agitation at 220 rpm for 10 days. After centrifugation, cell pellets were washed twice and resuspended in sterile water. Supernatant was vacuum-filtrated through sterilized Whatman No. 1 paper to obtain cell-free culture filtrate. Six treatment groups were set up in three replicates with seven seedlings for each: (a) Non-inoculated control 1 where plants were irrigated with sterile water; (b) Non-inoculated control 2 where plants were irrigated with blank culture medium; (c) Foc TR4 inoculated control; (d) Foc TR4 inoculation followed by treatment with 15 mL XY006 cell suspensions in water (10^6^ CFU/mL); (e) Foc TR4 inoculation followed by treatment with 15 mL XY006 cell-free culture filtrate; (f) Foc TR4 inoculation followed by treatment with 15 mL XY006 whole fermentation culture. For pathogen inoculation, banana roots were slightly wounded and immersed in Foc TR4 spore suspensions (10^7^ conidia/mL) for 12 h before the seedlings were transplanted to the pots. In groups d-f, the application of XY006 cell suspensions or fermentation culture to banana roots was conducted right after Foc TR4 inoculation. Foc TR4 was inoculated only once while strain XY006 application treatments were applied every 7 days for up to 45 days. Disease symptoms were then recorded using a five-point rating scale previously described (Zou et al., 2021). Disease incidence (%), disease severity index (%), and biocontrol efficiency (%) were calculated according to the literatures (Fan et al., 2021; Zou et al., 2021).


$$\begin{array}{c} \mathrm{Disease}\\ \mathrm{incidence}\;\left(\%\right) \end{array}=\mathrm{no}.\mathrm{of diseased plants}/\mathrm{total}\;\mathrm{no}.\mathrm{of plants}\times \mathrm{100}.$$

Disease severityindex%=Σno.of diseased plantsatalllevels×representative value of the level/totalno.of plants×representative value of the highest level×100.

Biocontrol efficiency%=control disease index−treatment disease index/control disease index×100.

### Quantification of the activities of antioxidant defense related enzymes in Foc TR4 infected banana plantlets following strain XY006 application

The antioxidant defense related enzymes, namely peroxidase (POD), catalase (CAT), superoxide mutase (SOD), polyphenol oxidase (PPO) and phenylalanine ammonia lyase (PAL) were quantified from Foc TR4 infected banana plantlets grown in the pot condition at 45 dpi with or without strain XY006 application.

Representative samples of roots and corms were collected from banana plantlets. Approximately 0.3 g of each sample was washed thoroughly with 1 mL of 0.9% saline buffer (pH 7.0) and ground in the same buffer using a pre-chilled mortar and pestle. After centrifugation, the supernatant was used as the crude enzyme extract. The enzyme activities were determined using commercial kits from Nanjing Jiancheng Bioengineering Institute, following the manufacturer’s protocols. All experiments were performed in triplicate and the enzyme activity was expressed as units per gram of fresh weight (FW).

### Evaluation of growth-promoting effects of strain XY006 on banana plantlets

The plant growth-promoting potential of XY006 on banana plantlets was evaluated under growth chamber conditions as described above. To prepare the inoculum, XY006 was cultured in MYG broth and incubated at 30°C with agitation at 220 rpm for 10 days. For the treatment, 10 mL XY006 culture (10^6^ CFU/mL) was used to drench banana plantlets, which was repeated once a week. For the control, the same amount of sterile water was applied. The experiment was conducted in sterilized pots (6.5 cm × 6.5 cm) containing sterilized soil. At 60 days, the plantlets were uprooted. Plant dry weights, root dry weights and lengths, stem perimeters as well as leaf chlorophyll contents and N contents were recorded. Five plantlets were used for each treatment and the treatment was performed in triplicate.

## Results

### Purification and structural characterization of antifungal compounds produced by strain XY006

**Figure 1:**
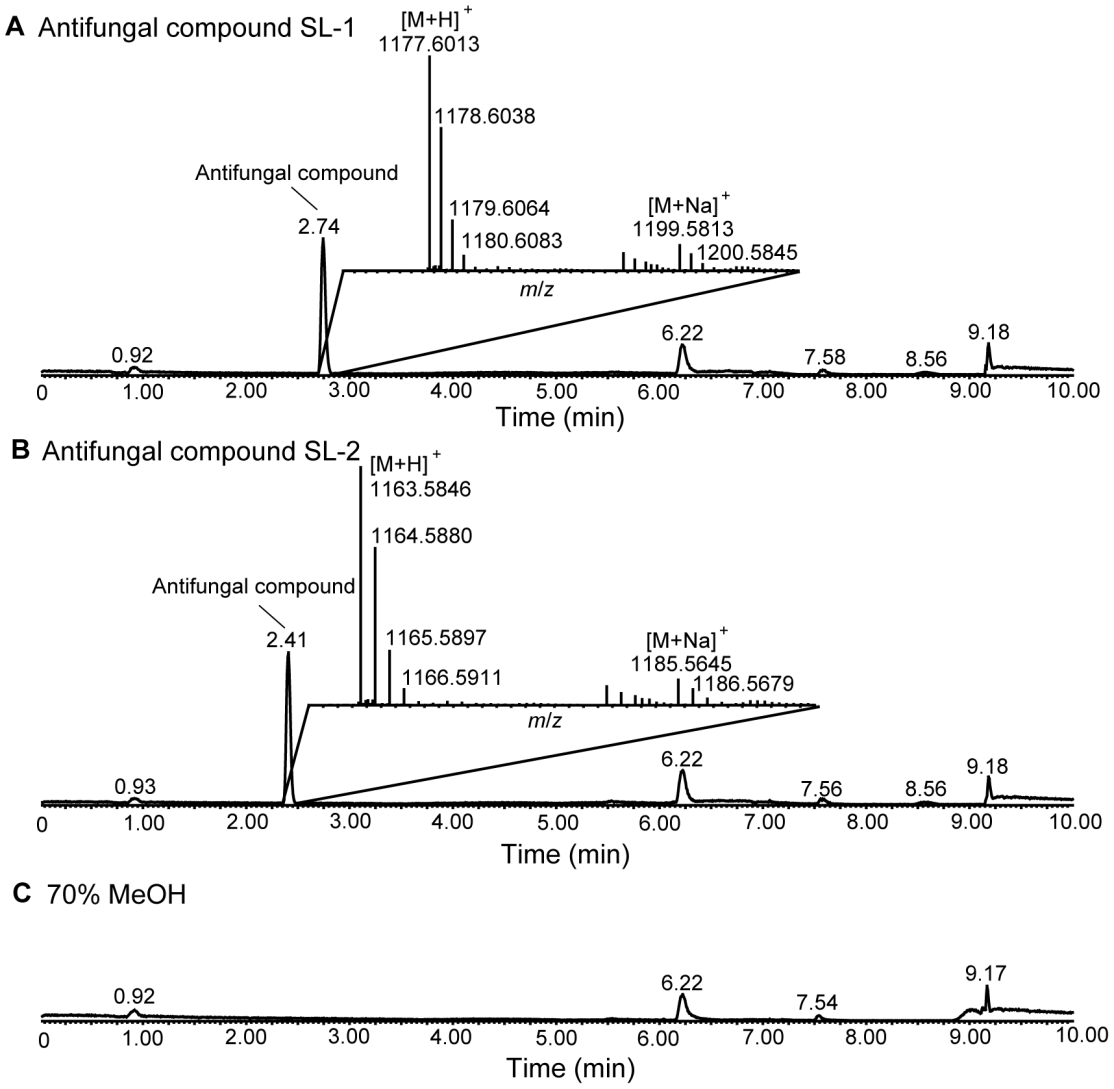
LC–MS analysis of the two antifungal compounds produced by strain XY006. **(A)** Total ion chromatogram (TIC) of the antifungal compounds eluted at 2.74 min. **(B)** TIC of the antifungal compounds eluted at 2.41 min. The insets show the MS spectra for these two metabolites. **(C)** TIC of 70% methanol solvent control. The peak labels in the spectrum are observed values.

The cell-free culture filtrate of strain XY006 was subjected to sequential methanol and *n*-butanol extraction, HLB solid phase extraction and reversed-phase HPLC to afford two antifungal compounds, namely SL-1 and SL-2. The purity of these two compounds, eluted as single peaks, was determined by UPLC-QTOFMS under positive electrospray ionization (ESI) mode. SL-1 eluted at 2.74 min gave the pseudomolecular ion peak at *m*/*z* 1177.6013 [M + H]^+^ and the correlated sodium adduct at *m*/*z* 1199.5813 [M + Na]^+^ (Figure 1A). The molecular formula was deduced as C_54_H_84_N_10_O_19_. SL-2 eluted at 2.41 min showed the pseudomolecular ion peak at *m*/*z* 1163.5846 [M + H]^+^, together with the peak for the sodium adduct at *m*/*z* 1185.5645 [M + Na]^+^ (Figure 1B). The molecular formula was established as C_53_H_82_N_10_O_19_. The molecular weight of SL-2 was 14 Da lower compared to SL-1, indicating the absence of a methylene group. Compared with 70% methanol solvent control, no other major peaks were detected in the chromatograms (Figure 1C). Thus, our purification scheme enabled the successful isolation of high-purity antifungal metabolites from strain XY006.

**Figure 2:**
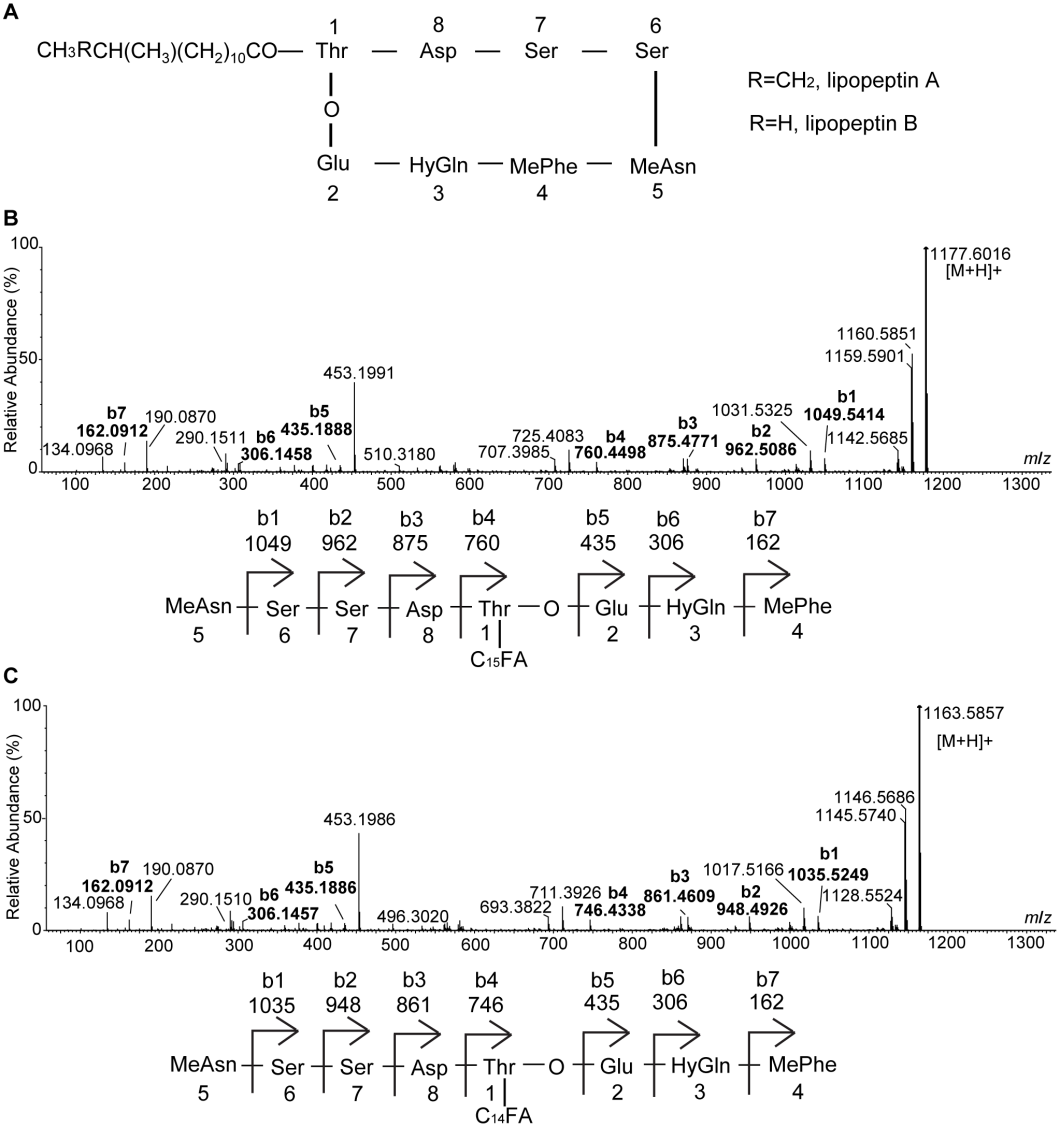
Electrospray ionization (ESI)-MS/MS (collision-induced association) spectra of the two antifungal compounds produced by strain XY006. **(A)** Chemical structures of lipopeptin A and lipopeptin B. **(B)** MS/MS spectrum of [M + H]^+^ ion at *m*/*z* 1177.6016 eluted at 2.74 min in Figure 1A. **(C)** MS/MS spectrum of [M + H]^+^ ion at *m*/*z* 1163.5857 eluted at 2.41 min in Figure 1B. The peak labels in the spectrum are observed values while predicted b series fragmentation ions are displayed at the bottom of each spectrum.

SL-1 and SL-2 have identical mass with lipopeptin A and lipopeptin B (Figure 2A), two cyclic lipopeptides that were initially characterized from a soil-dwelling *Streptomyces* isolate from Japan (Nishii et al., 1980; Tsuda et al., 1980). To gain more structural insights, MS/MS analyses were performed on the precursor ions at *m*/*z* 1,177 and 1,163. In the ESI-MS/MS spectrum of the precursor ion at *m*/*z* 1,177, a series of b^+^ fragment ions at *m*/*z* 1,049 → 962 → 875 → 760 → 435 → 306 → 162 were observed, consistent with the consecutive losses of MeAsn (L-methylasparagine), Ser, Ser, Asp., C_15_ fatty acid chain-Thr, Glu, HyGln (*β*-hydroxyglutamine) and MePhe (L-methylphenylalanine) residues from the *C*-terminal end (Figure 2B). Product ion spectrum of *m/z* 1,163 showed a series of similar b^+^ fragments at *m*/*z* 1,035 → 948 → 861 → 746 → 435 → 306 → 162, consistent with the consecutive losses of MeAsn, Ser, Ser, Asp., C_14_ fatty acid chain-Thr, Glu, HyGln and MePhe residues from the *C*-terminus (Figure 2C). Notably, the fragment ions of 435, 306 and 162 were common to SL-1 and SL-2 while 14 Da mass differences were present only in fragments carrying the fatty acid chain. Therefore, the two antifungal metabolites had the same amino acid sequence. Based on the MS/MS results, the linear sequence for SL-1 and SL-2 was proposed: fatty acid chain-Thr-Asp-Ser-Ser-MeAsn-MePhe-HyGln-Glu with cyclization between Thr and Glu. This was in agreement with the reported structures of lipopeptin A and lipopeptin B (Nishii et al., 1980).

### Effect of combined lipopeptide treatment on growth, membrane components and pathogenicity of Foc TR4

**Figure 3:**
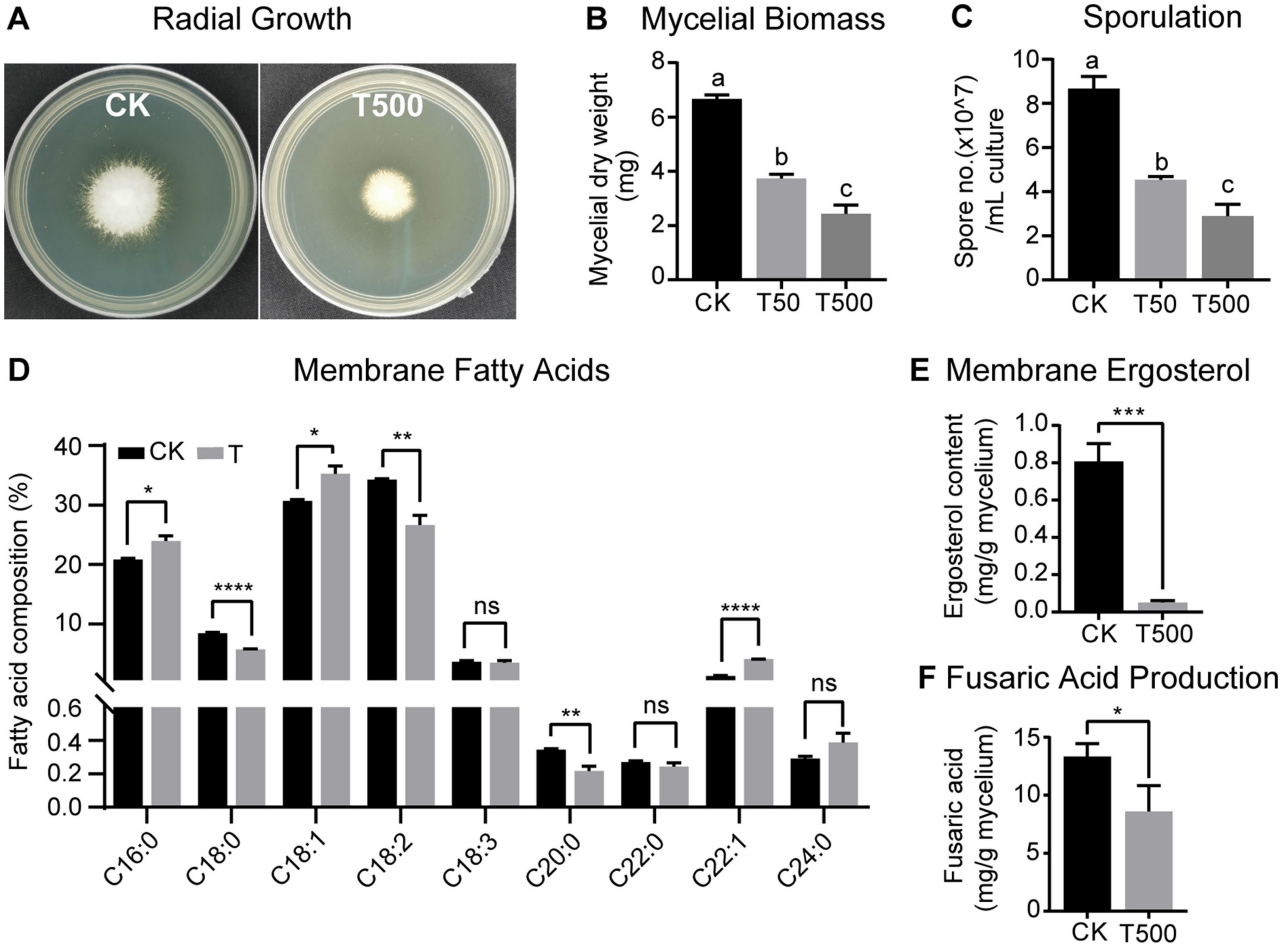
Antagonistic effects of combined lipopeptin A and lipopeptin B treatment on radial growth **(A)**, mycelial biomass **(B)**, sporulation **(C)**, membrane fatty acid content **(D)**, membrane ergosterol content **(E)**, and fusaric acid production **(F)** in Foc TR4 cells. CK, T50 and T500 represent treatment with 50 μg/mL blank culture medium, 50 μg/mL, and 500 μg/mL purified lipopeptides, respectively. Values are presented as mean ± SD (*n* ≥ 3). Different lowercase letters indicate statistically significant differences among treatments according to Tukey’s HSD test at *p* < 0.05. * Indicates significant differences between two groups (*t*-test, * *p* < 0.05, ** *p* < 0.01, *** *p* < 0.001, **** *p* < 0.0001). ns = not significant.

Due to the limited amounts of individually purified metabolites, lipopeptin A and lipopeptin B complex (referred hitherto as “lipopeptides”) with a combined purity >90% (see Supplementary Figure S1) was used to evaluate the antifungal activity against Foc TR4. The results demonstrated that the mixture of the two compounds at the concentration of 500 μg/mL inhibited the radial growth of Foc TR4, leading to 47.5% reduction in the mycelium diameter following 3 days of treatment (Figure 3A). Lipopeptides inhibited both mycelial growth and conidial sporulation in a dose-dependent manner, showing pronounced inhibition at even 50 μg/mL concentration (Figures 3B,C). The maximal effect was obtained in the 500 μg/mL lipopeptide treatment group, where mycelial dry weight and spore production declined by 63.5% and 66.5%, respectively.

Membrane disruption is the most commonly reported bioactivity associated with antifungal peptides (Yeaman and Yount, 2003; Rautenbach et al., 2016). To test this, the variations in Foc TR4 membrane fatty acid and ergosterol concentrations in response to combined lipopeptide treatment were quantified by GC-TOFMS. The palmitic acid (C16:0), stearic acid (C18:0), oleic acid (C18:1), linoleic acid (C18:2), and linolenic acid (C18:3) comprised the major fatty acids of the Foc TR4 lipid profile (Figure 3D). Following 24 h incubation with lipopeptides, a drastic reduction (by 42.8%) in the total membrane fatty acid content was observed. In terms of the relative abundance of individual fatty acids, fungus treated with lipopeptides showed a higher content of palmitic acid (C16:0) and oleic acid (C18:1) while a lower content of stearic acid (C18:0) and linoleic acid (C18:2), albeit the ratio of unsaturated/saturated fatty acid stayed relatively constant between the treatment and the control groups (Figure 3D). In addition, lipopeptide treatment drastically lowered the total content of ergosterol, a vital cell membrane component in fungi (Figure 3E). Results from the above analyses indicated that Foc TR4 cell membrane integrity was compromised after the exposure to lipopeptides.

To determine the effect on the production of FA, which is an important *Fusarium* pathogenicity factor, lipopeptides were supplemented to the Foc TR4 culture grown in Czapek broth medium and FA content was recorded at 15 dpi. According to GC–MS analysis, the addition of lipopetides lowered FA production in the culture by 35.5% compared to the control (Figure 3F). This likely implied that lipopeptides produced by strain XY006 could degrade or suppress the synthesis of FA.

### Effect of combined lipopeptide treatment on the ultrastructure of Foc TR4

**Figure 4:**
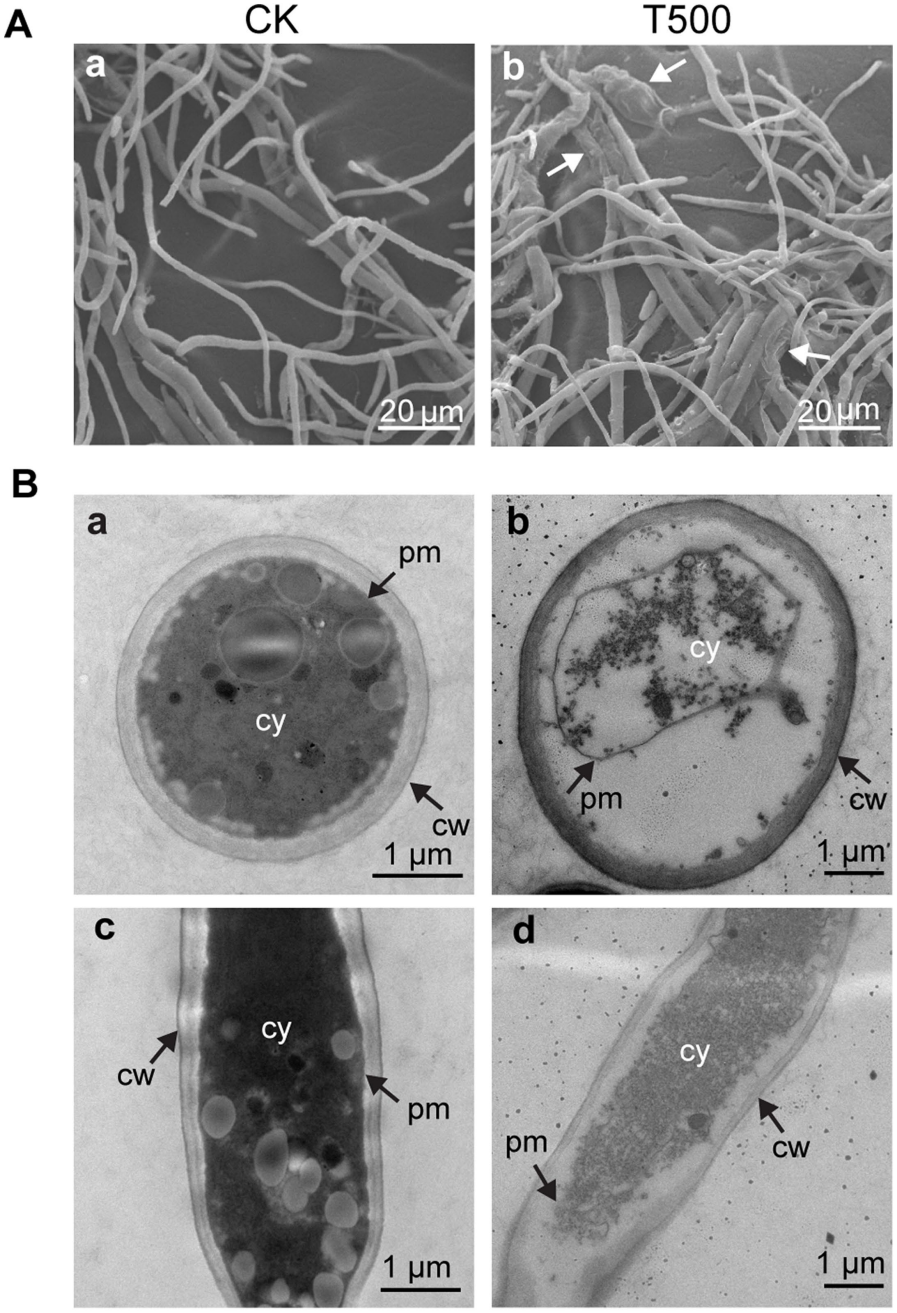
Effect of combined lipopeptin A and lipopeptin B treatment on the morphology and ultrastructure of Foc TR4 cells observed by SEM **(A)** and TEM **(B)**. CK, Foc TR4 hyphal cells treated with 50 μg/mL blank culture medium (a and c in panels **A**,**B**). T500, Foc TR4 hyphal cells treated with 500 μg/mL purified lipopeptides (b and d in panels **A**,**B**). The arrows in panel A indicate damaged mycelia of Foc TR4 in the presence of lipopeptides. cw, cell wall; pm, plasma membrane; cy, cytoplasm.

SEM and TEM were used to further demonstrate the morphology and ultrastructure changes of Foc TR4 hyphal cells. The morphology of Foc TR4 mycelia without lipopeptides showed normal growth with smooth surface, whereas the mycelia grown in the presence of lipopeptides appeared swollen and roughened (Figure 4A). TEM observation showed that the untreated Foc TR4 showed a well-defined cell wall, complete plasma membrane, and uniform and dense cytoplasm (Figures 4B,a,c). In contrast, the mycelia exposed to lipopeptides had a degraded cell wall, shrunk plasma membrane and unevenly distributed cytoplasm with empty areas, indicating that the cell membrane was severely disrupted (Figures 4B,b,d).

### Comparative antifungal activity of lipopeptin A and lipopeptin B against Foc TR4

**Figure 5:**
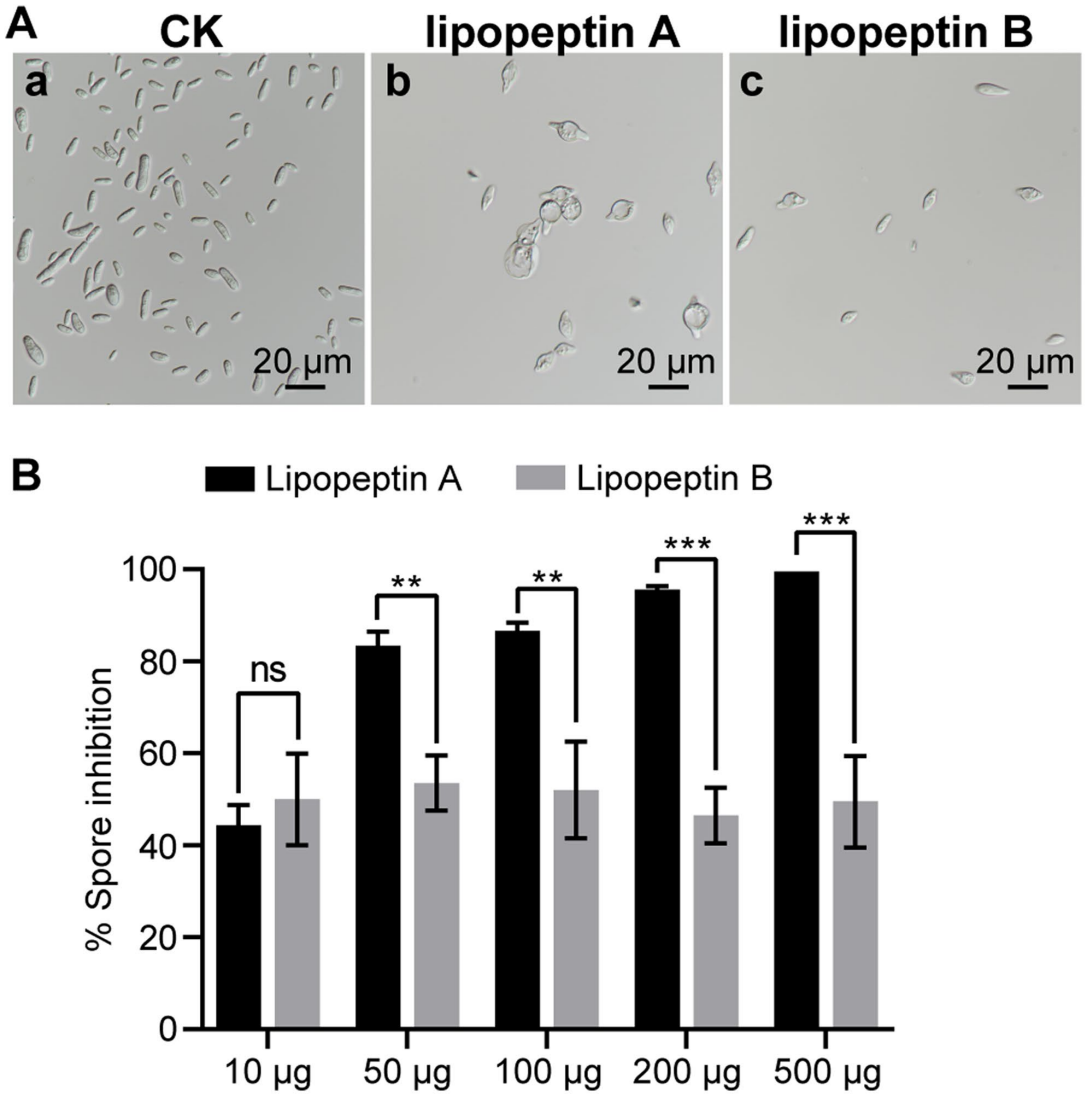
Comparative antifungal effect of lipopeptin A and lipopeptin B on spore morphology **(A)** and sporulation **(B)** of Foc TR4 cells. CK, lipopeptin A and lipopeptin B in panel **(A)** represent Foc TR4 spores treated with 50 μg/mL blank culture medium, 50 μg/mL lipopeptin A, and 50 μg/mL lipopeptin B, respectively. The percentage of spore inhibition was calculated relative to CK. Values are presented as mean ± SD (*n* ≥ 3). ^*^ Indicates significant differences between two groups (*t*-test, ^*^p < 0.05, ^**^p < 0.01, ^***^p < 0.001). ns = not significant.

Lipopeptin A and lipopeptin B differ by only one methylene group in their fatty acid chains. To investigate whether there is a difference in the antifungal activities, their effects on Foc TR4 sporulation and spore morphology were compared. When applied at 50 μg/mL, both lipopeptin A and lipopeptin B treatment gave rise to distorted and swollen morphology of Foc TR4 spores, with lipopeptin A displaying a more pronounced effect (Figure 5A). Lipopeptin A exhibited notably higher inhibition rates against fungal sporulation than lipopeptin B at nearly all tested doses ranging from 10 to 500 μg (Figure 5B). Moreover, the observed inhibition was proportional to the concentration of lipopeptin A, indicating a clear dose–response relationship. Conversely, lipopeptin B only showed moderate activity, regardless of the concentrations applied (Figure 5B). The calculated EC50 values for lipopeptin A and lipopeptin B were 11.12 μg/mL and > 500 μg/mL, respectively. Therefore, the antifungal activity of lipopeptin A was significantly higher than that of lipopeptin B.

### Colonization and distribution of transformed strain XY006 in banana plantlets

**Figure 6:**
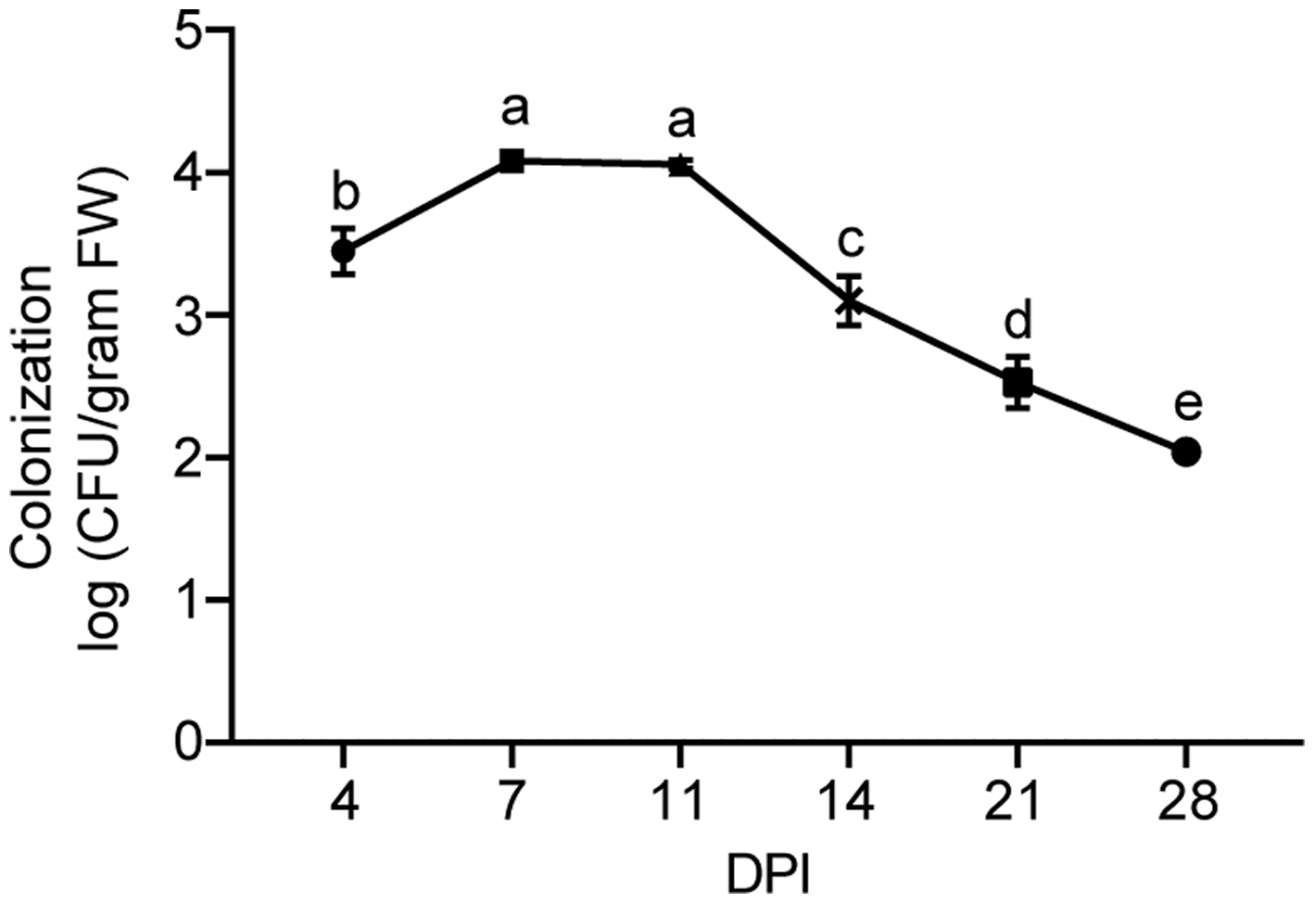
Population dynamics of strain XY006 residing in banana corm tissues. Values are presented as mean ± SD (*n* = 5). Different lowercase letters indicate statistically significant differences among treatments according to Tukey’s HSD test at *p* < 0.05. DPI, day post inoculation.

To study the plant colonization dynamics of strain XY006, we followed its population size in the inner tissues of banana plantlets over time. For this purpose, we transformed strain XY006 with pIB139 plasmid harboring the cloned *eGFP* gene under the control of a strong constitutive promoter and apramycin resistance by conjugation. The *eGFP* gene was detected in the positive transformants (see Supplementary Figure S2). In addition, the transformed XY006 (t-XY006) showed the same mycelium growth rate and antifungal activity as the wild type, indicating that gene transformation did not affect the strain fitness (see Supplementary Figure S3). Unfortunately, only very weak green fluorescence was observed by fluorescence microscopy (data not shown). As such, apramycin resistance was alternatively used to re-isolate the t-XY006 strain after plant inoculation. The results showed that this strain could be recovered from the surface sterilized roots and corms but not leaves (see Supplementary Figure S4). Dilution plating followed by CFU counts was used as a measure of the total quantity of t-XY006 colonizing banana plantlets. Since the t-XY006 strain was outgrown by other root-residing bacteria on the cultivation plate (see Supplementary Figure S4), quantification of this strain in roots was not performed. In corms, the cell counts of t-XY006 initially increased for the first 7 days post inoculation, stayed relatively stable until day 11, and then decreased gradually up to the end of the experiment at day 28 (Figure 6). The success of root and corm colonization suggests a promising potential of strain XY006 for Fusarium wilt disease management in bananas.

### Growth promotion and biocontrol activity of strain XY006 on banana plantlets in pot experiments

**Figure 7:**
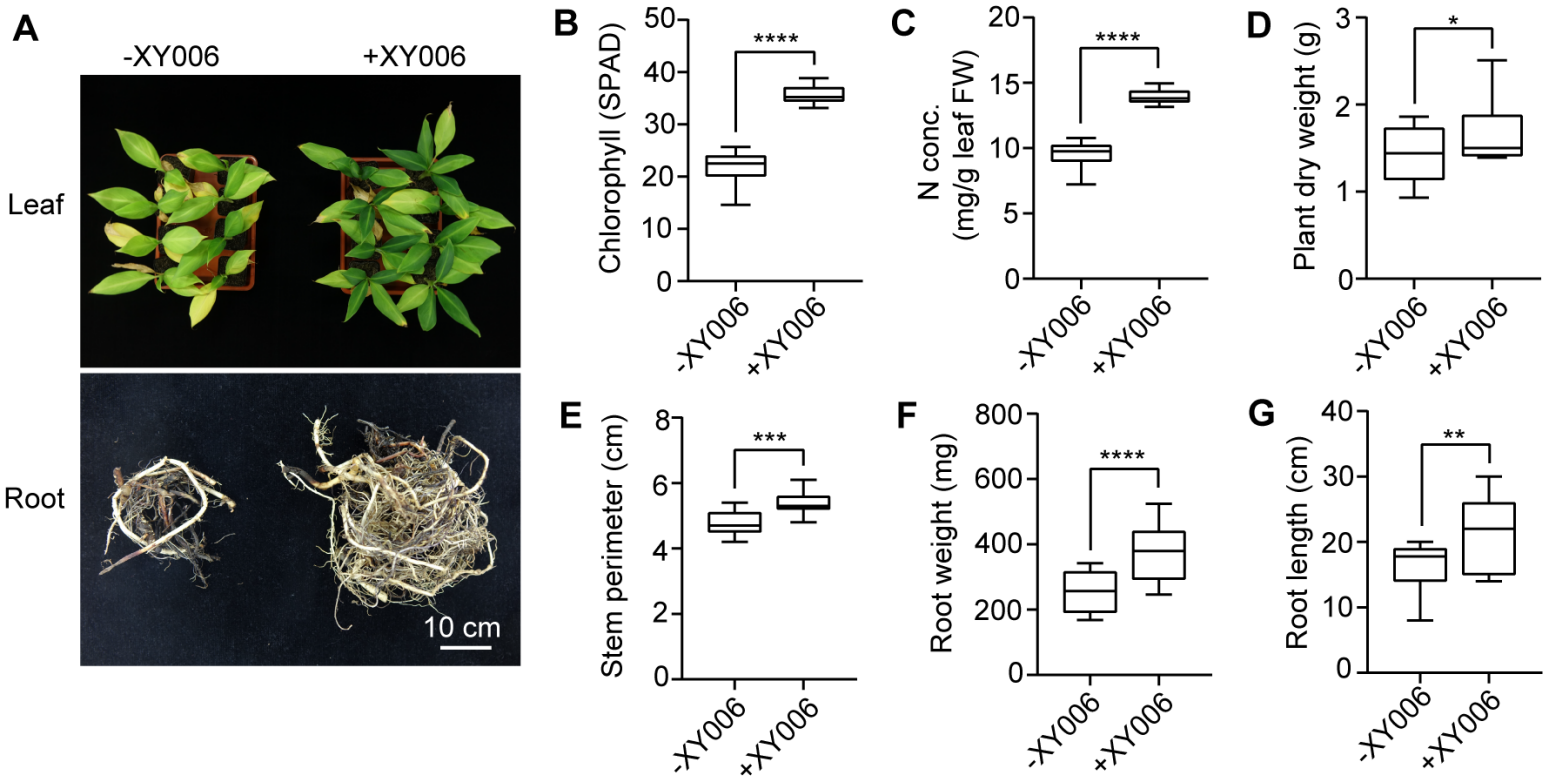
Plant growth promoting effect of banana seedlings with and without strain XY006 inoculation. **(A)** Leaf and root phenotypes of banana seedlings treated with strain XY006 after a two-month growing period in pots. Bar = 10 cm. **(B)** Leaf chlorophyll content (SPAD value). **(C)** Leaf nitrogen content. **(D)** Plant dry weight. **(E)** Stem perimeters. **(F)** Root weight. **(G)** Root length. Values are presented as mean ± SD (*n* ≥ 3). ^*^ Indicates significant differences between two groups (*t*-test, ^*^p < 0.05, ^**^p < 0.01, ^***^p < 0.001, ^****^p < 0.0001).

We next investigated whether strain XY006 could promote growth in banana plantlets. At 60 dpi, inoculation of banana plantlets with XY006 resulted in remarkable differences in terms of leaf morphology and root biomass (Figure 7A). A noteworthy increase in all tested parameters such as chlorophyll content, leaf nitrogen content, plant dry weight, stem perimeter, root weight and root length was noted in XY006-inoculated plants as compared to the mock-inoculated control (Figures 7B–G).

**Figure 8:**
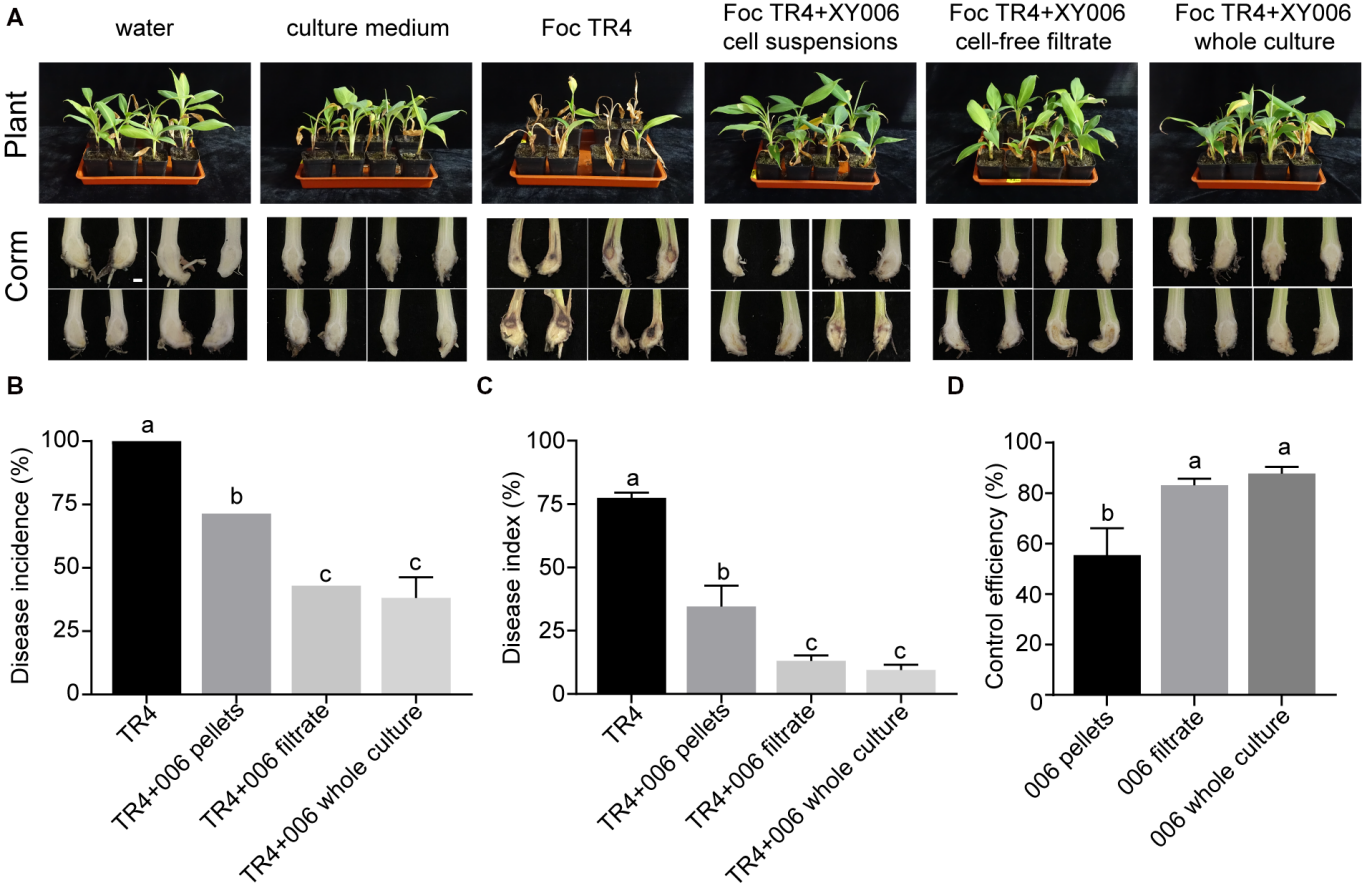
Performance of strain XY006 in the control of FWB. **(A)** Growth phenotypes of banana seedlings at 60 days post strain inoculation. Bar = 1 cm. **(B)** Statistical analysis of wilt disease incidences. **(C)** Statistical analysis of wilt disease indices. Five-scale was used to assess disease severity. **(D)** Control efficiency of XY006 fermentation culture against FWB. Values are presented as mean ± SD (*n* ≥ 3). Different lowercase letters indicate statistically significant differences among treatments according to Tukey’s HSD test at *p* < 0.05.

Additionally, the biocontrol efficacy of strain XY006 against FWB was examined in pot experiments. Banana plantlets treated with XY006, either the whole culture, cell-free filtrate or cell suspensions, showed much milder disease symptoms than the Foc TR4 alone treatment group (Figure 8A). Of note, the disease reduction was most prominent when the whole culture was applied, leading to much-reduced disease incidence and severity index relative to the control (Figures 8B,C). The biocontrol efficacy of this group reached 87.7%, demonstrating that strain XY006 has an excellent suppressive effect on Fusarium wilt (Figure 8D).

### Biochemical changes in Foc TR4 infected banana plantlets induced by strain XY006 application

**Figure 9:**
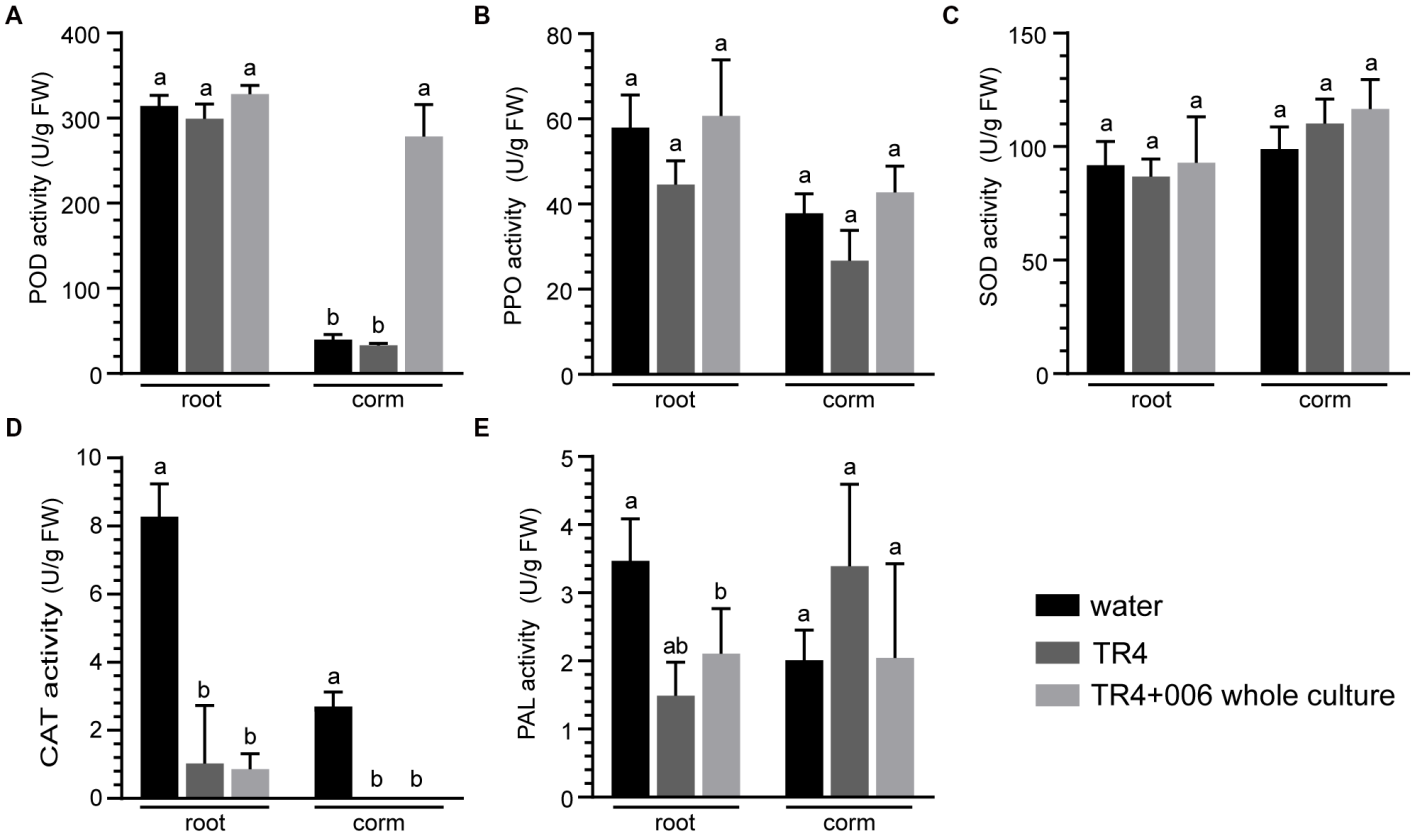
Biochemical changes in Foc TR4 infected banana plantlets following strain XY006 application. **(A)** POD activity. **(B)** PPO activity. **(C)** SOD activity. **(D)** CAT activity. **(E)** PAL activity. Different lowercase letters indicate statistically significant differences among treatments according to Tukey’s HSD test at *p* < 0.05.

Finally, to evaluate whether the disease tolerance of banana plantlets against Foc TR4 improved as a result of induced defense response, we measured the activities of five antioxidant enzymes (POD, CAT, SOD, PPO, and PAL) in the roots and corms of Foc TR4 infected banana plantlets with or without strain XY006 application. The results showed that strain XY006 had a different effect on the activity of these enzymes (Figure 9). At 45 dpi, the inoculation of strain XY006 resulted in a significant increase (7–8 fold) of POD activity in corms compared to negative controls in the pot condition (Figure 9A). However, there were no marked changes observed in the activities of PPO, SOD, CAT and PAL in either roots or corms following the inoculation of strain XY006 (Figures 9B–E).

## Discussion

FWB caused by Foc has posed a severe threat to banana production worldwide, with Foc TR4 being the most virulent race (Butler, 2013; Ghag et al., 2015). Efforts have been made by many breeding programs towards developing banana cultivars resistant to Fusarium wilt (Damodaran et al., 2009; Gonçalves et al., 2019). In spite of this, no commercial varieties that are fully Foc TR4-resistant are yet available (Damodaran et al., 2020). Soil fumigation using chemical fungicides (e.g., carbendazim) is a conventional agronomic practice to control soil-borne pathogens, but their extended use has raised environmental, economic and safety concerns, and has occasionally led to the emergence of fungicide resistance (Getha and Vikineswary, 2002; Cannon et al., 2022). While crop rotation can effectively reduce FWB incidence (Wang et al., 2015; Fan et al., 2022), this method is not always practical for large-scale banana plantations due to the extended period of time it requires. In this context, the prospect of developing and deploying biocontrol strategies, which serves as a sustainable and eco-friendly means for managing Fusarium wilt, has been extensively explored.

Biocontrol using microorganisms against Fusarium wilt has become a research hot spot in recent years (Raza et al., 2017; Bubici et al., 2019). These microbes compromise plant pathogens directly by producing antibiotics or lytic enzymes, or indirectly by inducing plant resistance or through nutrient competition (Bubici et al., 2019). *Trichoderma, Pseudomonas*, *Bacillus* and non-pathogenic *Fusarium* species are among the most evaluated BCAs for controlling FWB (Raza et al., 2017). *Streptomyces* spp., being the most prolific antibiotic producers, are somewhat less explored for their effectiveness in managing FWB, despite that this group of microorganisms has been well regarded as excellent BCAs of various phytopathogens (Viaene et al., 2016; Bubici, 2018). For instance, Zhou et al. reported that a rhizospheric *Streptomyces* isolate inhibited mycelial growth and spore germination of Foc TR4 by 85.4% and 80.1%, respectively; it reduced the incidence of FWB by 73% in pot experiments (Zhou et al., 2017). Also in pot trials, *Streptomyces* sp. CB-75 was effective against FWB, exhibiting 83.1% control efficacy and good plant growth promotion effect (Chen et al., 2018). A recent study conducted in the greenhouse condition revealed that infection of Foc TR4 in the roots and corms of banana seedlings was much suppressed by the application of *Streptomyces* sp. SCA3-4, a strain isolated from the rhizosphere soil of *Opuntia stricta* (Qi et al., 2022). Collectively, these studies indicate that *Streptomyces* are promising candidates for the biocontrol of FWB, although existing studies have been mostly confined to *in vitro* analyses, with only a few being conducted *in planta* (Bubici, 2018).

The antifungal activities of *Streptomyces* are largely attributed to the production of diverse assortments of antimicrobial metabolites (Barka et al., 2015). Nevertheless, identifying key metabolites that potentially confer biocontrol activity is challenging given that many *Streptomyces* strains used as biological control agents are capable of producing multiple specialized metabolites. In this study, by isolation, purification and functional characterization, we showed that two cyclic lipopeptides, namely lipopeptin A and lipopeptin B produced by strain XY006 (Figures 1, 2) exert potent antifungal activity towards Foc TR4. Combined treatment with lipopeptin A and lipopeptin B drastically inhibited mycelial growth and sporulation in Foc TR4 by targeting ergosterol synthesis and disturbing fatty acid homeostasis, leading to the weakening of cell membrane, leakage of the cellular contents and eventually loss of fungal viability (Figures 3A–E, 4).

Lipopeptin A and lipopeptin B were initially isolated in 1980 by Tsuda and coworkers from *Streptomyces* sp. no. AC-69 of a soil sample as a major and a minor component, respectively (Tsuda et al., 1980). Lipopeptin A displayed only weak inhibitory activity against some species of phytopathogenic fungi (*Piricularia oryzae*, *Colletotrichum lagenari*um, *Alternaria mali*, *Botrytis cinera*, and *Cochliobolus miyabeanus*) by inducing the swelling of mycelia, whereas the bioactivity of lipopeptin B was not described. Additionally, mice tolerated intravenous injection of 250 mg/kg of lipopeptin A, suggesting that this compound has a low toxicity (Tsuda et al., 1980). A later mechanistic study revealed that lipopeptin A inhibited cell wall (especially peptidoglycan) synthesis in *P. oryzae* possibly by blocking the transfer of *N*-acetylglucosamine into peptidoglycan (Nishii et al., 1980). Since then, no further information concerning the mode of action of these two compounds has been reported. In this study, we showed that the antifungal activity of lipopeptin complex is largely based on the direct targeting of the fungal cell membranes. In fact, membrane-targeting is integral for the antimicrobial activity of many lipopeptides (Rautenbach et al., 2016). For example, polymyxins, the last resort drugs to treat infections caused by multi-drug resistant Gram-negative bacteria, exert anti-microbial activity by perturbing cellular membranes (Sengupta et al., 2013). Other well-known examples are three antimicrobial lipopeptide families produced by *Bacillus* spp., namely surfactin, fengycin and iturin, acting primarily *via* membrane disruption (Zhao et al., 2017). Therefore, the membrane activity of lipopeptin complex observed from this study is consistent with those reported for many amphipathic lipopeptide antibiotics. Fusaric acid (FA) is known to induce wilt symptoms in a wide variety of plant species and hence has an essential role to play in plant pathogenesis (Singh et al., 2017). We found that FA production in Foc TR4 was suppressed by lipopeptin exposure (Figure 3F), hinting that certain enzymes in the biosynthetic pathway of this phytotoxin may be inactivated. The potential to inhibit FA synthesis possibly suggests an additional mode of action apart from causing membrane dysfunction. This is fascinating since antibiotics interacting with multiple targets could decrease the possibility of inducing fungal resistance. Our results add a new prospect to the antifungal potential of lipopeptin A and lipopeptin B, and prove that they deserve more exploration to delineate their mechanisms of action.

As typical for lipopeptides produced by microorganisms (Chooi and Tang, 2010; Zhang et al., 2023), lipopeptin A and lipopeptin B were also isolated predominantly as congener mixtures. They possess the same cyclic octapeptide core formed *via* an ester bond between L-Thr and L-Glu, and differ only in the *N*-terminal fatty acyl chain, which is branched with a chain length of 14–15 carbon atoms (Nishii et al., 1980). Despite the high degree of structural similarity, the separation of lipopeptin A and lipopeptin B was successfully accomplished by two rounds of reversed-phase HPLC, yielding >90% pure compounds (Figure 1) well suited to evaluate the structure–activity relationships. Although the structural difference between lipopeptin A and lipopeptin B is minimal, there is a significant difference in their potency against Foc TR4, with the former being much more potent (Figure 5). This again confirms the notion that variations in the lipid side chains have significant impacts on the bioactivities of lipopeptides (Chooi and Tang, 2010). Detailed investigations into the structure–activity relationships are required to decipher the optimal acyl chain length desired for maximal anti-Foc TR4 activity. In this regard, it necessitates the identification and study of the biosynthetic gene clusters involved in the synthesis of lipopeptin A and lipopeptin B in strain XY006 so that functional manipulation would be possible to generate lipopeptide variants with enhanced antifungal properties.

The competence for host colonization is essential for beneficial interactions between the plant and the biocontrol agent (Hardoim et al., 2015). We showed that by root dipping, XY006 isolated endophytically from the tea plant could colonize the roots and corms of banana plantlets (see Supplementary Figure S4). In other colonization studies, bacterial endophytes have proved their abilities to colonize a wide range of plants beyond their host plants (Kandel et al., 2017; Bubici et al., 2019). Within the corm tissue, strain XY006 was detected as early as 4 dpi and the degree of colonization reached the maximum at 7 dpi; although the population of this strain tended to decrease over time, it remained detectable at 28 dpi (Figure 6). Colonization of strain XY006 appeared to be restricted only to the lower part of the plant, as it was not recovered from the surface-sterilized banana leaves post inoculation. Since the experiments only lasted for less than a month after strain inoculation, the possibility that strain XY006 could make its way into leaves in longer colonization could not be eliminated, though this needs to be further determined. The successful colonization by strain XY006 led to increased growth and nutrition of banana plantlets in comparison to the mock-inoculated control (Figure 7). Strain XY006 possesses multiple plant growth promoting features, such as indole-3-acetic acid production (Shan et al., 2018), 1-aminocyclopropane-1-carboxylic acid deaminase activity (Shan et al., 2018) and phosphate solubilization (data not shown), likely accounting for the positive growth response observed in banana plantlets.

The antifungal property of strain XY006 was further demonstrated in this study under pot experiments. Application of this strain as the whole fermentation culture, cell-free culture filtrate or cell suspensions by soil drenching all drastically reduced disease incidence and severity index compared to Foc TR4-infected plants (Figure 8). In particular, banana plantlets treated with XY006 whole fermentation culture showed the lowest disease incidence caused by Foc TR4 (62% incidence reduction). Chitin and *β*-glucans are the predominant components of fungal cell walls. Besides antifungal metabolites, the production of siderophores (Sayyed et al., 2013) and hydrolytic enzymes such as chitinases (Neeraja et al., 2010), glucanases (Guo et al., 2013) and proteases (Singh and Chhatpar, 2011) are also strategies that beneficial bacteria employ to target pathogenic fungi. In the XY006 genome, there are eight putative chitinase genes, seven putative glucanase genes, 67 putative protease genes and three putative siderophore biosynthetic gene clusters (data not shown). They may act synergistically to contribute to the biocontrol efficacy of XY006. In addition, it is known that plants exposed to BCAs and subsequently infected with pathogens exhibit increased production of antioxidant enzymes such as SOD, catalase and peroxidase and this elevated enzyme activity provides protection against oxidative damage and cell death in plants (Martinez et al., 1998). The results of our study indicated a significant alternation in the activity of POD in the corms of banana plantlets treated with strain XY006 at 45 dpi (Figure 9). A time-course analysis of the interactions between the banana plantlets and strain XY006 would provide a detailed understanding of the dynamics of the plant-microbe interaction over time, allowing for a comprehensive assessment of the effect of strain XY006 on the disease resistance of banana plantlets.

Collectively, this study highlights the potential of strain XY006 as a promising candidate for controlling FWB, which is largely attributed to the production of two lipopeptide antibiotics, lipopeptin A and lipopeptin B. These two lipopeptides, with lipopeptin A exhibiting a higher antifungal activity, antagonize Foc TR4 by inducing membrane perturbation, inhibiting conidia sporulation and mycelial growth, and reducing fusaric acid production. While this study provides interesting insights into the mechanisms underlying the biocontrol activity of XY006, further investigation into strain colonization and its mechanism of action is needed to deepen the understanding of the tritrophic interactions between the host plant, the pathogen and the endophyte. Moreover, long-term field studies with a much larger sample size are essential to assess the efficacy of strain XY006 in the biological control of FWB, taking into account the potential limitations of using a small number of plants in our experiment.

## Data availability statement

The original contributions presented in the study are included in the article/Supplementary material, further inquiries can be directed to the corresponding authors.

## Author contributions

XY and ZW conceived and designed the experiments. XW, ZD, CC, SG, QM, WW, RW, WH, PX, YZ, and WS performed the experiments. XW analyzed the data. XW, XY, and ZW interpreted the results. XY and XW wrote the manuscript. All authors contributed to the article and approved the submitted version.

## Funding

This work was funded by the National Natural Science Foundation of China (32002093) and the Fujian Agriculture and Forestry University (FAFU) Construction Project for Technological Innovation and Service System of Tea Industry Chain (K1520005A02).

## Conflict of interest

The authors declare that the research was conducted in the absence of any commercial or financial relationships that could be construed as a potential conflict of interest.

## Publisher’s note

All claims expressed in this article are solely those of the authors and do not necessarily represent those of their affiliated organizations, or those of the publisher, the editors and the reviewers. Any product that may be evaluated in this article, or claim that may be made by its manufacturer, is not guaranteed or endorsed by the publisher.
