# Supplementary material for: Antifungal effects and biocontrol potential of lipopeptide-producing Streptomyces against banana Fusarium wilt fungus Fusarium oxysporum f. sp. cubense
Source: Front Microbiol. 2023 Apr 27;14:1177393. doi: 10.3389/fmicb.2023.1177393 (PMC10172682; doi:10.3389/fmicb.2023.1177393)
Supplement: Supplementary file 1 [file Data_Sheet_1.PDF]

Supplementary information

Antifungal effects and biocontrol potential of lipopeptide-producing *Streptomyces* against banana Fusarium wilt fungus *Fusarium oxysporum* f. sp. *cubense*

Xiaxia Wang[1,2]#, Zhenghua Du[2,3], Chanxin Chen[2,3], Shuang Guo[2,4], Qianzhuo Mao[5], Wei Wu[5], Ruimei Wu[2], Wenbo Han[2], Peifeng Xie[2], Yiping Zeng[2], Wenna Shan[2], Zonghua Wang[1,6]*, Xiaomin Yu[2]*

1. State Key Laboratory of Ecological Pest Control for Fujian and Taiwan Crops, College of Plant Protection, Fujian Agriculture and Forestry University, Fuzhou, China
2. FAFU-UCR Joint Center for Horticultural Biology and Metabolomics, Haixia Institute of Science and Technology, Fujian Agriculture and Forestry University, Fuzhou, China
3. School of Life Sciences, Fujian Agriculture and Forestry University, Fuzhou, China
4. College of Horticulture, Fujian Agriculture and Forestry University, Fuzhou, China
5. State Key Laboratory for Managing Biotic and Chemical Threats to the Quality and Safety of Agro-products, Institute of Plant Virology, Ningbo University, Ningbo, China
6. Fujian Universities Engineering Research Center of Marine Biology and Drugs, Fuzhou Institute of Oceanography, Minjiang University, Fuzhou, China

***Correspondence:**

Dr. Zonghua Wang (wangzh@fafu.edu.cn)

Dr. Xiaomin Yu (xmyu0616@fafu.edu.cn)

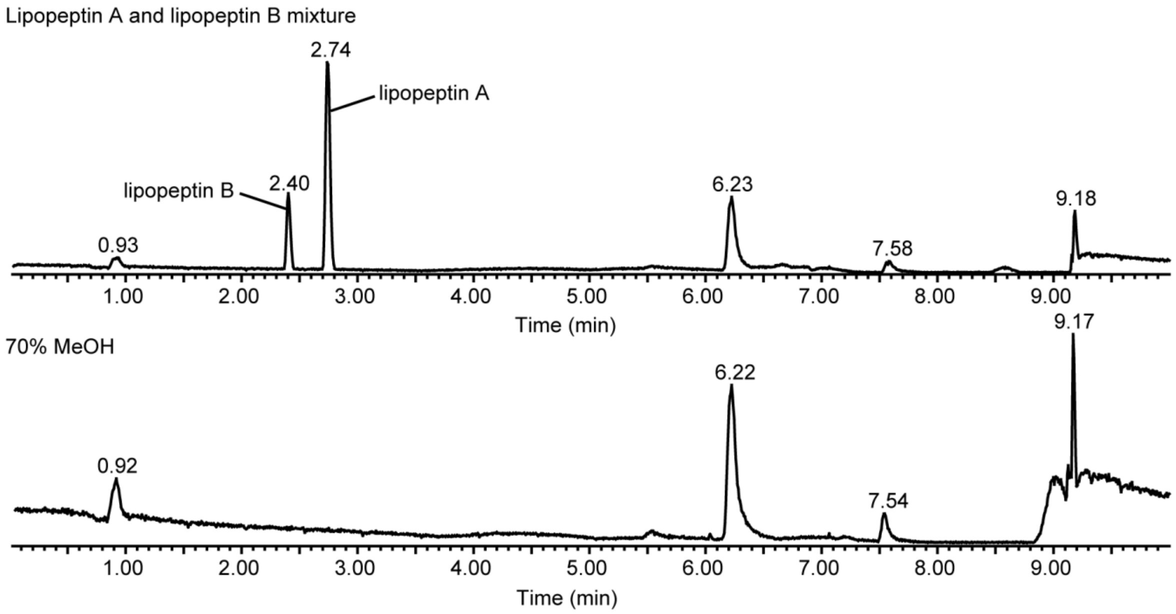

Figure S1. Total ion chromatogram (TIC) of one purified HPLC fraction comprising lipopeptin A and lipopeptin B. This fraction was used to study the antifungal effect on *Fusarium oxysporum* f. sp. *cubense* tropical race 4 (Foc TR4).The co-purified mixture of lipopeptin A and lipopeptin B has a combined purity > 90% as determined in comparison to the solvent control (bottom).

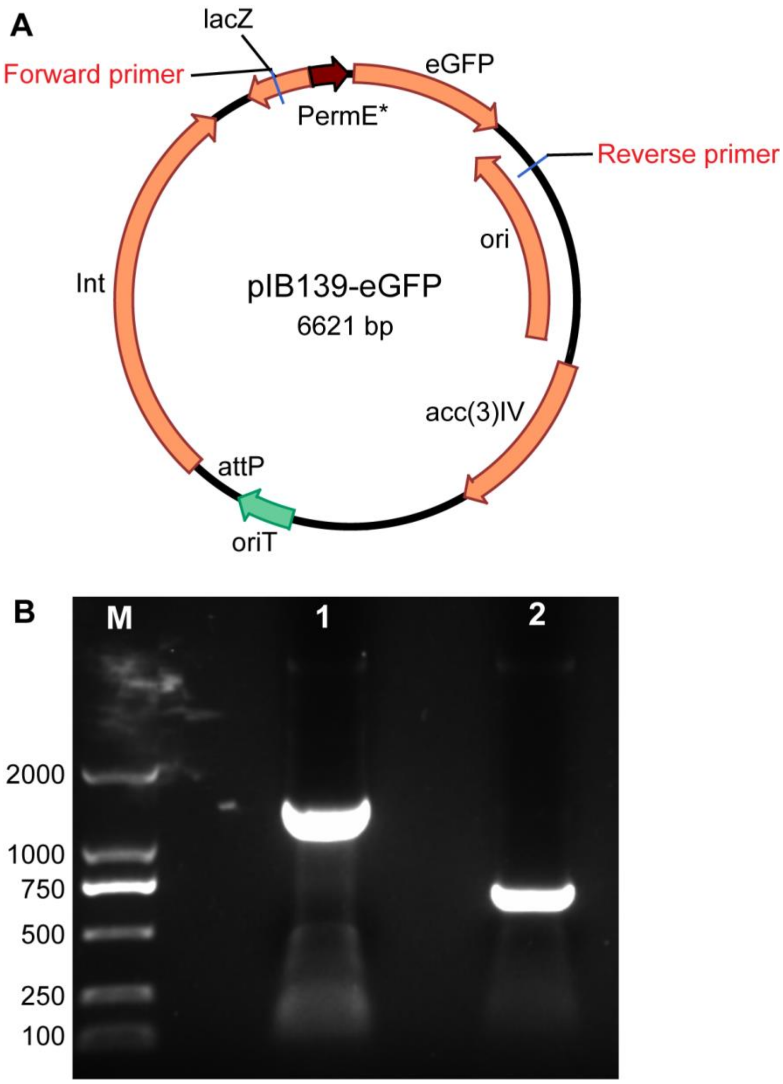

Figure S2. Verification of *eGFP* gene integration into the genome of *Streptomyces* sp. XY006. (A) Schematic illustration of the recombinant plasmid pIB139-*eGFP*. The binding sites of primers used for *eGFP* gene amplification were illustrated. (B) Gel electrophoresis of PCR fragments. Lane M, DNA marker; lane 1, gene amplification from the genomic DNA of pIB139-*eGFP*-transformed XY006; lane 2, gene amplification from the genomic DNA of pIB139-transformed XY006.

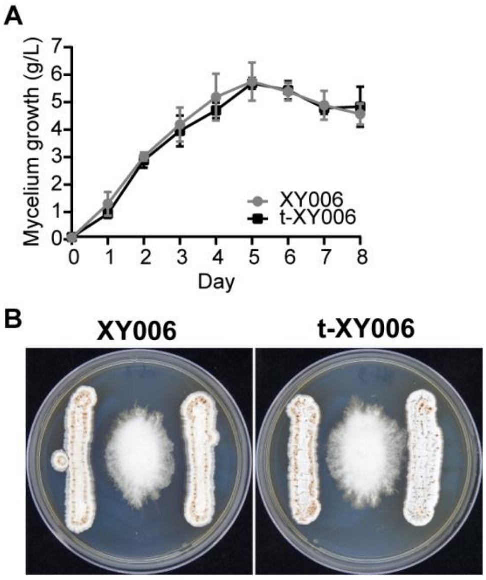

Figure S3. Mycelium growth (A) and antifungal activity (B) of the wild type XY006 and transformed XY006 against Foc TR4. Error bars represent standard deviations (n=3).

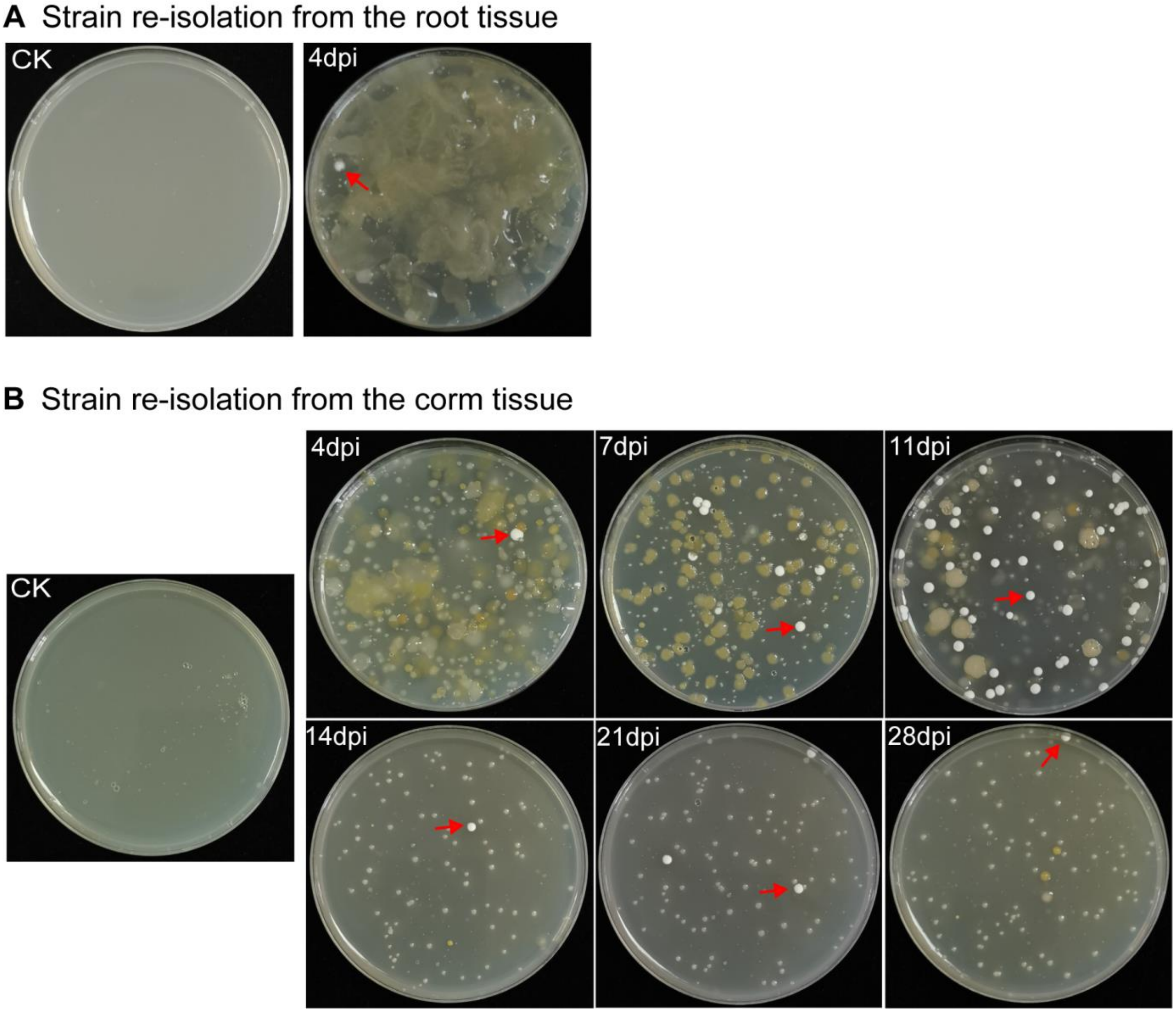

Figure S4. Strain re-isolation from surface-disinfected root (A) and corm (B) tissues of inoculated banana plantlets using the dilution plating method. Red arrows indicate the colony of *eGFP*-tagged strain XY006 as confirmed by 16S rRNA sequencing. The final rinse with sterile water after disinfection procedures was plated as the negative control to rule out the possible contamination from the plant surface.
